# A combined protein toxin screening based on the transcriptome and proteome of *Solenopsis invicta*

**DOI:** 10.1186/s12953-022-00197-z

**Published:** 2022-09-21

**Authors:** Liuyang Cai, Fengling Yang, Yongfang Wang, Jishun Yang, Yina Zhu, Xueqi Ma, Juan Höfer, Yichao Wang, Yajun Ma, Liang Xiao

**Affiliations:** 1 Basic School of Medicine, Second Military Medical University (Naval Medical University), Shanghai, 200433 China; 2 College of Traditional Chinese Medicine, Jilin Agricultural University, Changchun, 130118 Jilin China; 3 Faculty of Naval Medicine, Second Military Medical University (Naval Medical University), Shanghai, 200433 China; 4 Medical Insurance Center, Navy Medical Center, Navy Medical Center of PLA, Shanghai, 200050 China; 5 Escuela de Ciencias del Mar, Pontificia Universidad Católica de Valparaíso, Valparaíso, 2340000 Región de Valparaíso Chile; 6 Department of Clinical Laboratory, Taizhou Central Hospital, Taizhou, 318000 Zhejiang China

**Keywords:** Ant, *Solenopsis invicta*, Transcriptome, Proteome, Toxin

## Abstract

**Background:**

Multi-omics technology provides a good tool to analyze the protein toxin composition and search for the potential pathogenic factors of *Solenopsis invicta*, under the great harm of the accelerated invasion in southern China.

**Methods:**

Species collection, functional annotation, toxin screening, and 3D modeling construction of three interested toxins were performed based on the successfully constructed transcriptome and proteome of *S*. *invicta*.

**Results:**

A total of 33,231 unigenes and 721 proteins were obtained from the constructed transcriptome and proteome, of which 9,842 (29.62%) and 4,844 (14.58%) unigenes, as well as 469 (65.05%) and 71 (99.45%) proteins were annotated against the databases of Gene Ontology and Kyoto Encyclopedia of Genes and Genomes, respectively. After comparing with the uniprot toxin database, a total of 316 unigenes and 47 proteins (calglandulin, venom allergen 3, and venom prothrombin activator hopsarin-D, etc.) were successfully screened.

**Conclusions:**

The update of annotations at the transcriptome and proteome levels presents a progression in the comprehension of *S*. *invicta* in China. We also provide a protein toxin list that could be used for further exploration of toxicity as well as its antagonistic strategy by *S*. *invicta*.

**Supplementary Information:**

The online version contains supplementary material available at 10.1186/s12953-022-00197-z.

## Introduction

*Solenopsis invicta* Buren (Hymenoptera, Formicidae, Myrmicinae, *Solenopsis*), originally distributed in the Parana River Basin in South America [1], was listed as one of the most destructive invasive alien organisms by the International Union for Conservation of Nature. With the development of trade and transportation industry, the invasion of *S*. *invicta* occurs increasingly frequently and causes more serious harmfulness [2–4]. *Solenopsis invicta* was discovered in Taiwan in 2003 [5], recorded in mainland of China in 2004 [6], and then rapidly spread to 12 provinces and 448 counties as reported by the Ministry of Agriculture and Rural Affairs of the People’s Republic of China (http://www.moa.gov.cn/nybgb/) in April 2021. Meanwhile, nine departments have implemented control measures in 2021 to stop the spread of *S*. *invicta* in China, emphasizing the urgency and significance of *S*. *invicta* study (http://www.moa.gov.cn/ztzl).

As reported, *S*. *invicta* has negative impacts on the aspects of agricultural production, ecological environment, economic construction, and human health [7]. For example, the economic loss caused by *S*. *invicta* invasion in China was estimated to total USD 25 billion [8]. Moreover, *S*. *invicta* was known to aggressive species that can quickly inject the target with the acidic venoms from their abdominal through stinging needles, causing a series of adverse symptoms [9, 10]. In addition to the cutaneous symptoms (generalized cutaneous or large local), some individuals could be allergic to tiny amounts of the toxins of *S*. *invicta*, even leads to systemic poisoning and anaphylactic shock [11–13].

The venom of *S*. *invicta* is mainly composed of water, insoluble alkaloids, and trace amounts of proteins [14]. Each *S*. *invicta* stinging delivers 10 ~ 100 ng of proteins and 0.04 ~ 0.11 µL of venom [15]. Among them, the alkaloids account for about 95% of the venom of *S*. *invicta*, which was responsible for cell necrosis, pain, and pustule reactions [16, 17], although only 0.01% protein causing anaphylactic reaction [18]. Growing researches have focused on the toxins of *S*. *invicta* especially for the four allergic toxins [12, 18–23] but few researches were performed to clarify the specific amount, species, and functions of toxins from the perspective of multi-omics joint analysis. Currently, only 72 ant venom peptides from 11 ant species (not including *S*. *invicta*) have been fully sequenced [24]. Additionally, only two transcriptomes of ant venom glands have been published to date, revealing novel information of potential proteins in the venom gland [25–27], what is extremely small comparing to snakes, cone snails, scorpions and spiders [24]. Furthermore, several new components of the venom gland were identified based on the transcriptome because not all potential transcripts are necessarily translated into proteins, so need to be further confirmed through proteomic techniques [28]. Therefore, the combined analysis of transcriptome and proteome approach provides strong support for the toxins screening of *S*. *invicta*, which was beneficial to reveal a considerably wider variety of *S*. *invicta* toxins than previously reported and shed insights on the molecular evolution of them.

## Materials and methods

### Ant collection

The *S*. *invicta* samples were collected alive from Shantou Waisha International Airport in Guangdong, China on March 18, 2021 and were then transported alive in a sealed cylinder.

### Transcriptome

#### RNA sequencing and pre-processing

Samples were equally divided into triplicate and loaded into three centrifuge tubes. Total RNA of *S. invicta* (*n* = 3) were extracted using Trizol reagent (Invitrogen, USA) following the manufacturer’s procedure. The total RNA quantity and purity were analyzed by Bioanalyzer 2100 and RNA 1000 Nano LabChip Kit (Agilent, USA) with RIN number > 7.0. Poly (A) RNA (*n* = 3) was purified from total RNA (5 µg) using poly-T oligo-attached magnetic beads with two rounds of purification. After it, the mRNA was fragmented into small pieces using divalent cations under elevated temperature. Then the cleaved RNA fragments were reverse-transcribed to create the final cDNA library in accordance with the protocol of the mRNA-Seq sample preparation kit (Illumina, USA), the average insert size for the paired-end libraries was 300 bp (± 50 bp). The quality control of sequencing library was carried out and the raw data was obtained by the Illumina Novaseq™ 6000 platform (LC Science, USA).

Firstly, the cutadapt [29] and perl scripts in house were used to remove the reads with adaptor contamination, low-quality bases and undetermined bases. Then the raw sequencing intensities were transformed as the raw read, and sequence quality was verified by using FastQC software (https://www.bioinformatics.babraham.ac.uk/projects/fastqc/) including the Q20, Q30, and GC content of the clean data. In order to ensure the quality of the follow-up information analysis, raw data were filtered and high-quality data (clean data) were generated. Trimmomatic 0.36 was used to remove the joints as well as the sequences with low-quality, and fractions with more than Q20 were considered as high-quality reads. The four criteria for evaluating quality including single base quality, base content distribution, GC content distribution, and sequence base quality.

#### De novo transcriptome assembly

After obtaining high-quality clean data, all downstream analysis were performed on this basis. De novo assembly of the transcriptome was performed through using Trinity 2.4.0 [30]. Trinity groups transcripts into clusters based on shared sequence content. Such a transcript cluster was very loosely referred to as a ‘gene’. The longest transcript in the cluster was chosen as the ‘gene’ sequence (aka Unigene).

#### Annotation of transcripts

To obtain gene expression annotation, all assembled unigenes were aligned against the NCBI nonredundant (NR) database (http://www.ncbi.nlm.nih.gov/protein/), SwissProt (http://web.expasy.org/docs/swiss-prot_guideline.html), Gene Ontology (GO, http://geneontology.org/), and Kyoto Encyclopedia of Genes and Genome (KEGG, http://www.kegg.jp/) using the new comparison software DIAMOND [31] with a threshold of Evalue < 0.00001.

### Proteome

#### Protein extraction and digestion

Samples were equally divided into triplicate and loaded into three centrifuge tubes. The proteins of *S*. *invicta* (*n* = 3) were extracted by using Protein Extraction Kit (Bangfei Bioscience, China), and the proteins were quantified by the Bradford method. Then the protein samples were mixed with 5 μL 1 M DTT (Genview, USA) and kept at 37 ℃ for 1 h. 20 μL 1 M IAA (Vetec, Brazil) was added and reacted at room temperature for 1 h in the darkness. After centrifugation, the supernatant was mixed with 100 μL UA (8 M urea (Sigma, Japan), 100 mM Tris–HCl (Amresco, USA), pH 8.0) solution, and then followed by centrifugation twice. After the supernatant was discarded, the pellet was resuspended in 100 μL 50 mM NH_4_HCO_3_ (Sigma, Japan) solution, and then followed by centrifugation triple. The extracted protein was mixed with Trypsin (Promega, USA) at the ratio of 50: 1 and digested at 37 °C for 16 h.

#### LC-MS/MS analysis

Each sample was first separated using the nanoliter flow HPLC system Easy nLC1000 (Sigma, Japan). After equilibrating the column with 95% buffer A (0.1% FA (Sigma, Japan)), samples were loaded from the autosampler to the C18 trap column (3 μm, 0.10 μm × 20 mm) (Thermo Scientific, USA) and separated by an analytical C18 column (1.9 μm, 0.15 μm × 120 mm) (Thermo Scientific, USA) at a flow rate of 600 nL/min. Primary parent ion scanning was performed in the orbitrap after peptide fragmentation. The scanning range was set to 300 ~ 1,400 (m/z), the Orbitrap resolution (Thermo Scientific, USA) was set to 120,000, the automatic gain control target was set to 5 × 10^5^, and the maximum injection time was set to 50 ms. HCD activation type was used in secondary spectrograms (ddMSnScan) with a Iontrap, a collision energy was 30% and the stepped collision energy was 5%. The dynamic exclusion time was set to 18 s. The spray voltages of the positive and negative ion mode mass spectrometers were set to 2,000 V and 600 V separately, and the spray temperature was 320 ℃ for peptides.

#### Database search and bioinformatics analysis

Each sample was separated by capillary HPLC system (Thermo Scientific, USA) and analyzed by mass spectroscopy using a Q-Exactive HF mass spectrometer (Thermo Scientific, USA). Produced raw data of the RAW file was conducted with Proteome Discoverer software (Thermo Scientific, USA). GO was analyzed on proteins including Biological Process (BP), Cell Component (CC), and Molecular Function (MF). The pathways annotation was conducted by the primary public database of KEGG. Toxins in the transcriptome and proteome were screened against the uniprot database through local Blast method. Multiple sequence alignment analysis was performed with BioEdit software 7.0.5.3 under default parameters. The phylogenetic tree was built by the MEGA 7 with the Neighbor-Joining method. The screened proteins were modeled by SWISS-MODEL and displayed by Discovery studio 4.5.

## Results

### Species annotation

**Fig. 1:**
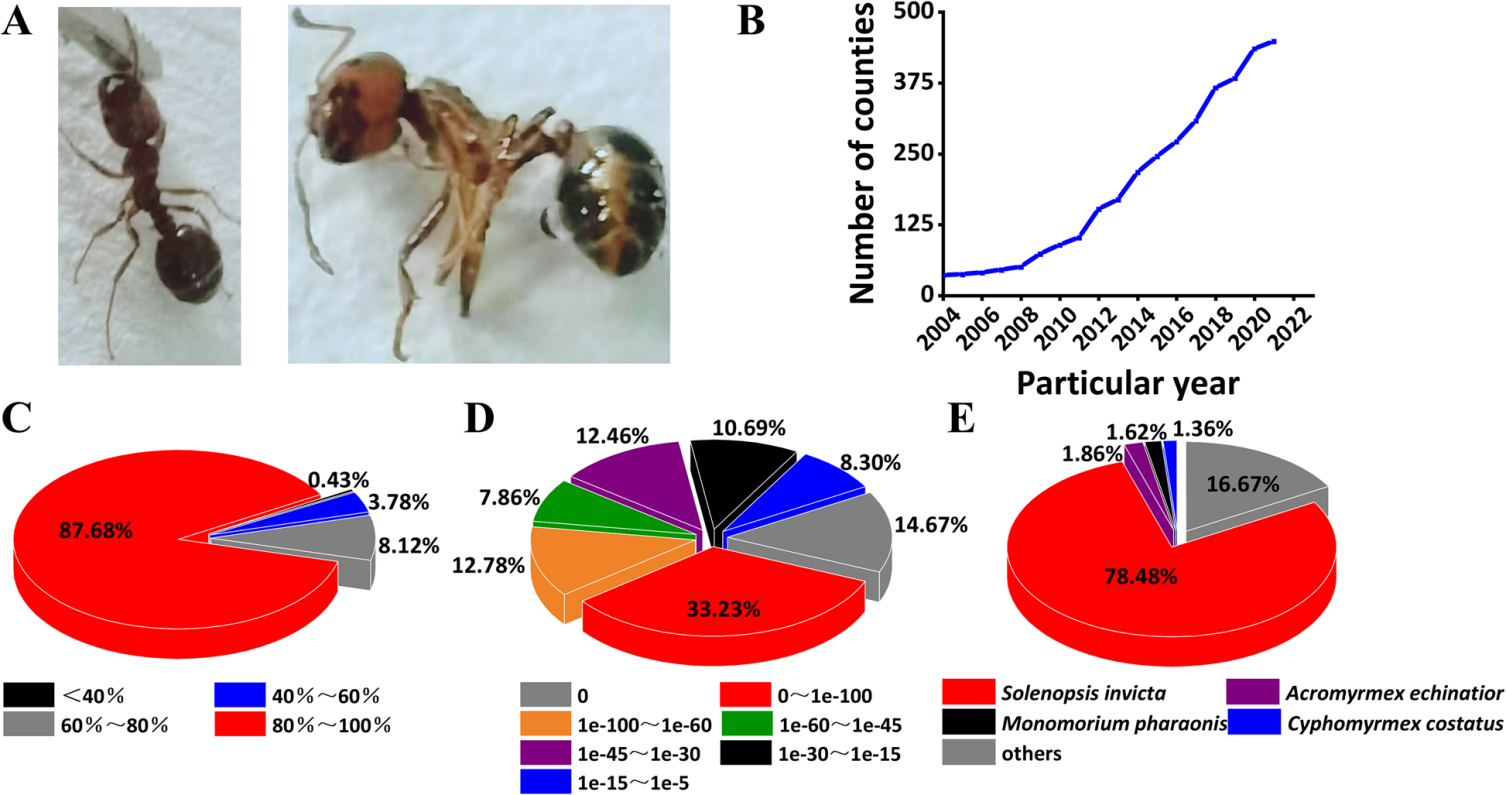
Species annotation. **A** *S*. *invicta* sample. **B** The increasing dynamics of the number of *S*. *invicta* in China. The y-axis was the number of counties with *S*. *invicta* occurrence, and the x-axis was the year. The data were obtained from the Ministry of Agriculture and Rural Affairs of the People’s Republic of China (http://www.moa.gov.cn/). **C** Similarity distribution. **D** E-value distribution. **E** NR species distribution

The *S*. *invicta* which have ten antennomeres with two club segments, copper brown in body and head, and dark in abdomen with sting, were growing massively in China and could be roughly divided into two stages (Fig. 1A and B). The period from the beginning of the invasion to 2008 was the initial stage of diffusion with the slow spread rate. After 2008, it entered the period of rapid diffusion (Fig. 1B). The rapid trend of *S*. *invicta* invasion has piqued our interest, whose transcriptome and proteome were also successfully constructed in our study. At the transcriptome level, the assembly quality of unigenes was evaluated by GC content and unigene length. The proteins of samples passed the quality inspection through evaluating the sequence coverage (%) distribution, peptide length distribution, peptide counts distribution, and the relative molecular weight distribution (Fig. S1). NR database showed the sequence with 80% ~ 100% similarity was the most abundant, accounting for 87.68% (14,203), followed by the sequence with 60% ~ 80% similarity, accounting for 8.12% (1315) (Fig. 1C). All the annotated sequences had low e-values, with the ranges of 0, 0 ~ 1e-100, 1e-100 ~ 1e-60, 1e-60 ~ 1e-45, 1e-45 ~ 1e-30, 1e-30 ~ 1e-15, and 1e-15 ~ 1e-5 accounting for 14.67%, 33.23%, 12.78%, 7.86%, 12.46%, 10.69%, and 8.30% of all sequences, respectively (Fig. 1D). Four species *S*. *invicta*, *Acromyrmex.echinatior*, *Monomorium.pharaonis*, and *Cyphomyrmex.costatus* were the most matched species, with the similarities of 78.48%, 1.86%, 1.62%, and 1.36%, respectively (Fig. 1E).

### Comparative GO analysis and KEGG pathways

**Fig. 2:**
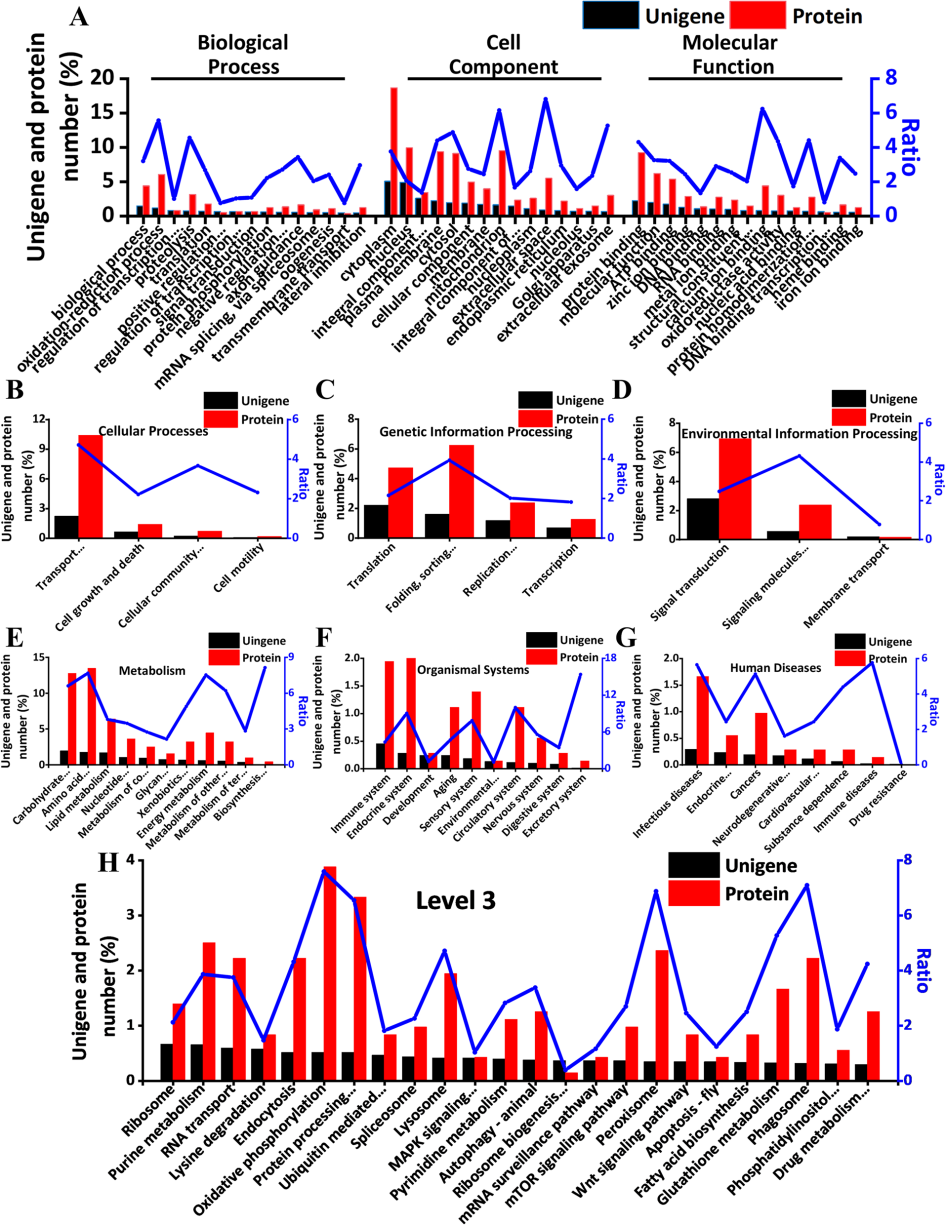
Comparative Gene Ontology (GO) analysis and KEGG pathways. **A** Comparative Gene Ontology (GO) analysis of unigenes and proteins. “regulation of transcription…”, “positive regulation…”, “regulation of transcription…”, “negative regulation…”, “integral component…”, “integral component of…”, “structural constituent…”, “protein homodimerization…”, and “DNA binding transcription…” in the figure actually represented as “regulation of transcription, DNA-templated”, “positive regulation of transcription by RNA polymerase II”, “regulation of transcription by RNA polymerase II”, “negative regulation of transcription by RNA polymerase II”, “integral component of membrane”, “integral component of plasma membrane”, “structural constituent of ribosome”, “protein homodimerization activity”, and “DNA binding transcription factor activity”, respectively. **B** Cellular processes. “Transport…” and “Cellular community…” in the figure actually represented as “Transport and catabolism” and “Cellular community—eukaryotes”. **C** Genetic information processing. “Folding, sorting…” and “Replication…” in the figure actually represented as “Folding, sorting and degradation” and “Replication and repair”. **D** Environmental information processing. “Signaling molecules…” in the figure actually represented as “Signaling molecules and interaction”. **E** Metabolism. “Carbohydrate…”, “Amino acid…”, “Nucleotide…”, “Metabolism of co…”, “Glycan…”, “Xenobiotics…”, “Metabolism of other…”, “Metabolism of ter…”, and “Biosynthesis…” in the figure actually represented as “Carbohydrate metabolism”, “Amino acid metabolism”, “Nucleotide metabolism”, “Metabolism of cofactors and vitamins”, “Glycan biosynthesis and metabolism”, “Xenobiotics biodegradation and metabolism”, “Metabolism of other amino acids”, “Metabolism of terpenoids and polyketides”, and “Biosynthesis of other secondary metabolites”, respectively. **F** Organismal systems. “Environmental…” in the figure actually represented as “Environmental adaptation”. **G** Human diseases. “Endocrine…”, “Neurodegenerative…”, and “Cardiovascular…” in the figure actually represented as “Endocrine and metabolic diseases”, “Neurodegenerative diseases”, and “Cardiovascular diseases”. **H** Level 3 KEGG pathways. “Protein processing…”, “Ubiquitin mediated…”, “MAPK signaling…”, “Ribosome biogenesis…”, “Phosphatidylinositol…”, and “Drug metabolism…” in the figure actually represented as “Protein processing in endoplasmic reticulum”, “Ubiquitin mediated proteolysis”, “MAPK signaling pathway—fly”, “Ribosome biogenesis in eukaryotes”, “Phosphatidylinositol signaling system”, and “Drug metabolism—other enzymes”, respectively

A total of 33,231 unigenes and 721 proteins were obtained from the successfully constructed transcriptome and proteome. Only 9,842 unigenes (29.62%) and 469 proteins (65.05%) were annotated against the GO database, and proteins had 2.20 times as the annotation percentage as unigenes. While only 4,844 unigenes (14.58%) and 717 proteins (99.45%) were annotated against the KEGG database, and proteins had 6.82 times as the annotation percentage of unigenes. Gene analysis of unigenes and proteins was performed by using the default parameters of Blast2GO software [32]. GO was classified into the following three categories: BP, CC, and MF. By selecting the unigene number in the top fifteen for mapping, we found that biological process and oxidation reduction process were the two richest terms in BP, with 451 (1.36%) and 358 (1.08%) in unigenes, and with 31 (4.30%) and 43 (5.96%) in proteins, respectively. Cytoplasm and nucleus were the two most abundant terms in CC, with 1656 (4.98%) and 1605 (4.83%) in unigenes, and with 134 (18.59%) and 71 (9.85%) in proteins, respectively. Extracellular space had the highest ratio in CC, with 6.78 times as the percentage of annotated proteins as unigenes. In MF, protein binding, molecular function and ATP binding were the three most abundant terms, with 711 (2.14%), 628 (1.89%) and 551 (1.66%) in unigenes, and with 66 (9.16%), 44 (6.10%), and 38 (5.27%) in proteins, respectively. Calcium ion binding had the highest ratio in MF, with 6.21 times as the percentage of annotated proteins as unigenes (Fig. 2A).

Unigenes and proteins were annotated against the KEGG database with the online automated annotation system KAAS (KEGG Automatic Annotation Server) (http://www.genome.jp/tools/kaas/). The pathways of KEGG were divided into six categories at the level 1 including cellular processes, genetic information processing, environmental information processing, metabolism, organismal systems, and human diseases. Among them, metabolism accounted for 10.18% in unigenes and 52.43% in proteins while human diseases accounted for only 1.10% in unigenes and 4.16% in proteins. At the level 2, in cellular processes, transport and catabolism were the most with 734 unigenes (2.21%) and 75 proteins (10.4%) annotated (Fig. 2B). In genetic information processing, 730 translations (2.20%) were annotated in unigenes, and 45 folding, sorting and degradation (6.24%) were annotated most in proteins (Fig. 2C). In environmental information processing, signal transduction was the most in both unigenes and proteins, with 931 (2.80%) and 50 (6.93%), respectively (Fig. 2D). Metabolism annotated the most abundant unigenes and proteins among the six categories (Fig. 2E). In organismal systems, excretory system had the largest ratio, and the ratio of the proteins was 15.36 times of the unigene annotation (Fig. 2F). In human diseases, infectious diseases were the most abundant, with 98 (0.29%) and 12 (1.66%) annotated in unigenes and proteins, respectively (Fig. 2G). The top one pathway with matched unigene was ribosome (218, 0.66%) whereas the most abundant in proteins was oxidative phosphorylation (28, 3.88%) (Fig. 2H).

### Potential toxins screening

**Fig. 3:**
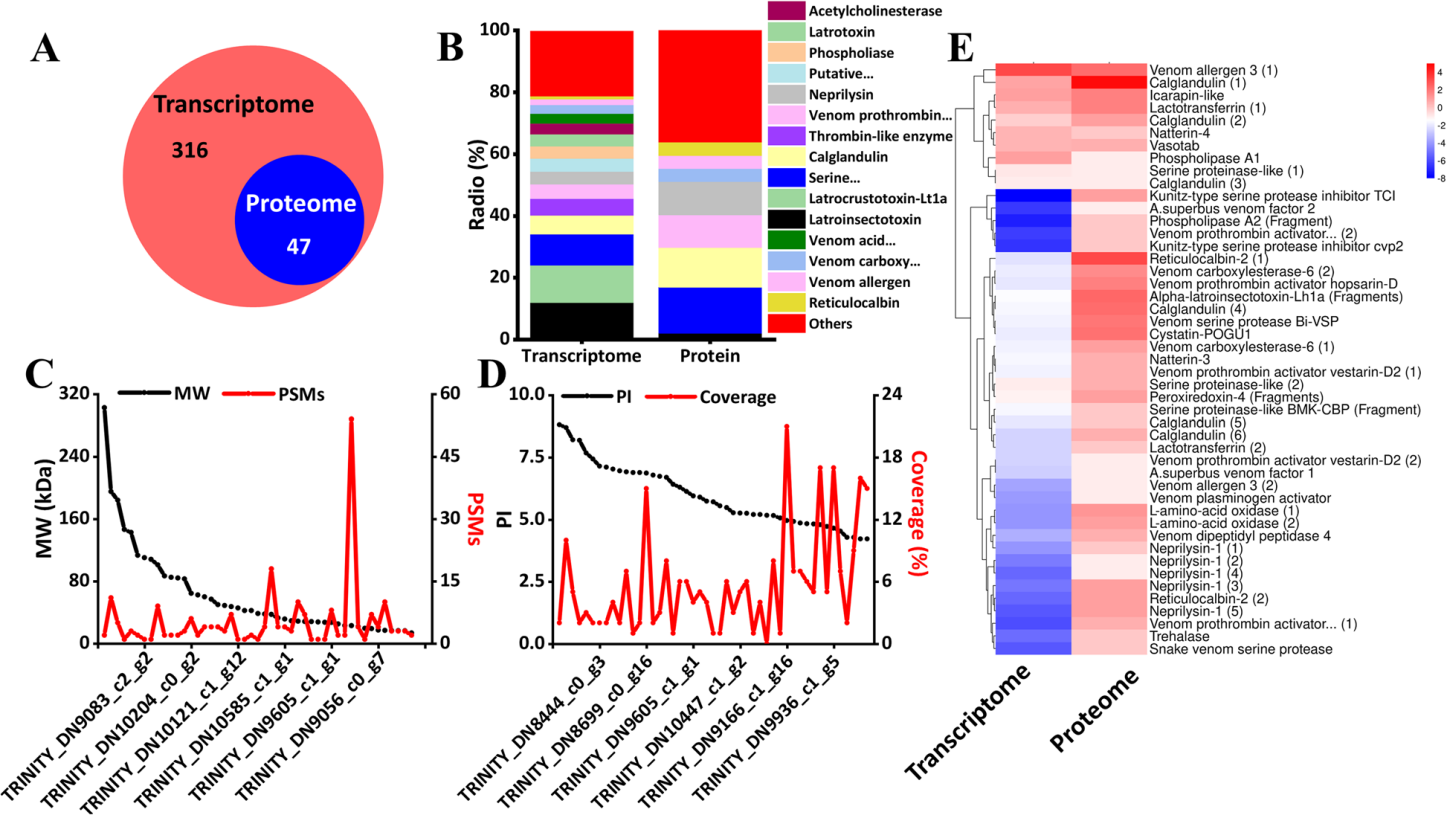
Potential toxins screening. **A** Potential toxins and enzymes were identified at the transcriptome and proteome levels of *S*. *invicta*. Red color and blue color represented transcriptome and proteome respectively. **B** Different colors represented different toxins or enzymes, and the size of the area represented the percentage of each toxin in the total. “Venom carboxy…”, “Venom acid…”, “Serine…”, “Venom prothrombin…” and “Putative…” stood for “Venom carboxylesterase-6”, “Venom acid phosphatase Acph-1”, “Serine proteinase/serine protease”, “Venom prothrombin activator”, and “Putative lysosomal acid lipase/cholesteryl ester hydrolase”, respectively. **C** The MW and PSMs of toxins. **D** The PI and amino acid coverage (Coverage) distribution of toxins. **E** The toxins were comparatively analyzed in transcriptome and proteome, all unigenes and proteins related to GAPDH2 were calculated by log_2_ (Fold change) and displayed by heatmap. “Venom prothrombin activator… (1)”, and “Venom prothrombin activator… (2)” represented as “Venom prothrombin activator omicarin-C non-catalytic subunit (1)”, and “Venom prothrombin activator omicarin-C non-catalytic subunit (2)”

A total of 316 and 47 toxin-related proteins were screened at the transcriptome and proteome levels through Blast annotation, respectively (Fig. 3A). At the transcriptome level, Latroinsectotoxin (12.03%), Latrocrustotoxin-Lt1a (12.03%), Serine proteinase/ serine protease (10.13%), Calglandulin (6.01%), Thrombin-like enzyme (5.38%), Venom prothrombin activator (4.75%), Neprilysin (4.11%), Putative lysosomal acid lipase/ cholesteryl ester hydrolase (4.11%), Phospholiase (4.11%), Latrotoxin (3.80%), Acetylcholinesterase (3.48%), Venom acid phosphatase Acph-1 (3.16%), Venom carboxylesterase-6 (2.85%), Venom allergen (1.90%), Reticulocalbin (0.95%), and Others (20.89%) were screened out. 47 proteins were further screened from 316 toxin-related uingenes. Among the toxins with the top fifteen highest richness in the transcriptome, only Latroinsectotoxin (2.13%), Serine proteinase/ serine protease (14.89%), Calglandulin (12.77%), Venom prothrombin activator (10.64%), Neprilysin (10.64%), Venom carboxylesterase-6 (4.26%), Venom allergen (4.26%), and Reticulocalbin (4.26%) were screened out in the proteome (Fig. 3B).

Characteristics of all toxins were analyzed including the molecular weight (MW), the peptide spectrum matches (PSMs), the isoelectric point (PI), and coverage (%). The MW of toxins were distributed between 13 ~ 303 kD, and the PSMs were mainly concentrated in 1 ~ 15 (Fig. 3C). The PI concentrated between 4.0 ~ 6.0 was accounting for 57.45%. The lowest coverage of amino acid in toxins was Venom prothrombin activator pseutarin-C non-catalytic subunit (TRINITY_DN9182_c1_g14, 0.00%), and the highest was Calglandulin (TRINITY_DN9166_c1_g16, 21.00%) (Fig. 3D). After it, we selected the toxins that both expressed at the transcriptome and proteome levels, and calculated their fold change with the expression of GAPDH2 which is a house-keeping gene. Among them, it showed that Venom allergen 3 (1) (TRINITY_DN8992_c2_g4) and Calglandulin (1) (TRINITY_DN9166_c1_g16) were both highly expressed at the transcriptome and proteome levels. In addition, Kunitz-type serine protease inhibitor TCI (TRINITY_DN8482_c2_g14), Phospholipase A2 (Fragment) (TRINITY_DN9050_c0_g3), Venom prothrombin activator omicarin-C non-catalytic subunit (1) (TRINITY_DN9341_c1_g13), and Snake venom serine protease (TRINITY_DN9155_c0_g11) were lowly expressed in the transcriptome while the opposite in the proteome (Fig. 3E).

### Calglandulin

**Fig. 4:**
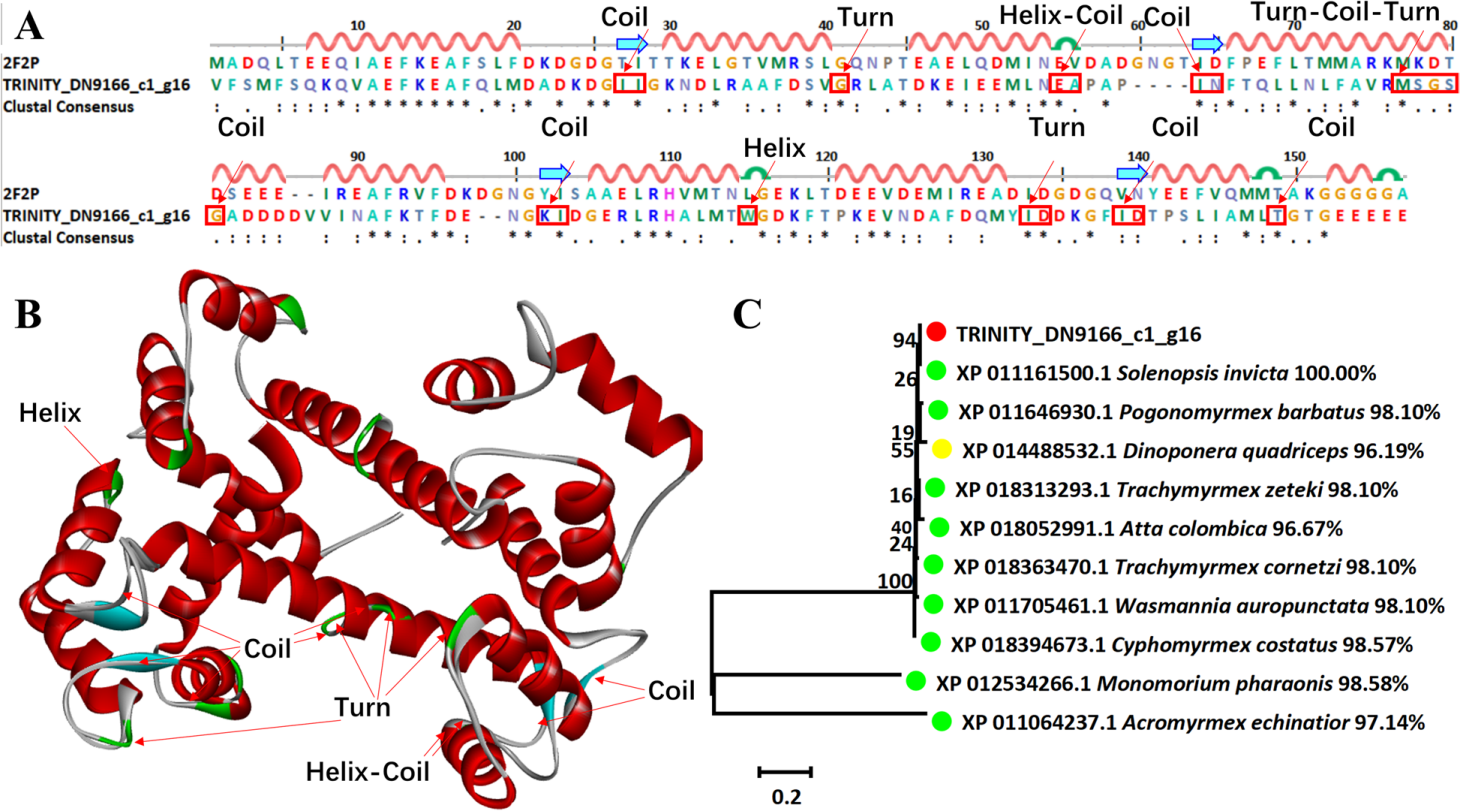
Sequence alignment, 3D modeling and phylogenetic analysis of Calglandulin. **A** Putative sequence TRINITY_DN9166_c1_g16 was aligned with the template (PDB ID: 2F2P). At the bottom of columns, asterisks (*) showed conserved positions, colons (:) showed conserved substitutions and points (.) showed non-conserved substitutions. Grey line, green bend, blue banded arrowhead, and red solenoid represent coil, turn, sheet, and helix, respectively. Different fragments were marked by red framework. **B** 3D modeling was modeled by SWISS-MODEL and viewed by Discovery Studio 4.5. The color grey, green, blue, and red represents coil, turn, sheet, and helix, respectively. The differences of TRINITY_DN9166_c1_g16 compared with the template (PDB ID: 2F2P) were marked with black font. **C** The phylogenetic tree including sequence TRINITY_ DN9166_c1_g16 and other ten sequences from different species was constructed by using MEGA 7 with the Neighbor-Joining method. Putative sequence, Myrmicinae, and Ponerinae were marked as red, green, and yellow circles, respectively

The dominate toxic component calglandulin was naturally performed sequence alignment and 3D modeling with a template (PDB ID: 2F2P) which showed the structure of calmodulin bound to a calcineurin peptide. Calglandulin belongs to the calmodulin family. TRINITY_DN9166_c1_g16 with 2F2P showed a similarity of 34.27%. There was a total of 14 differences between TRINITY_DN9166_c1_g16 and the template, which were also showed in the 3D structure, including 8 coils, 4 turns, and 2 helixes (Fig. 4A and B). The construction of phylogenetic tree intuitively showed close genetic relationship with formicidae and the highest similarity was *S*. *invicta* (100.00%) (Fig. 4C).

### Venom allergen 3

**Fig. 5:**
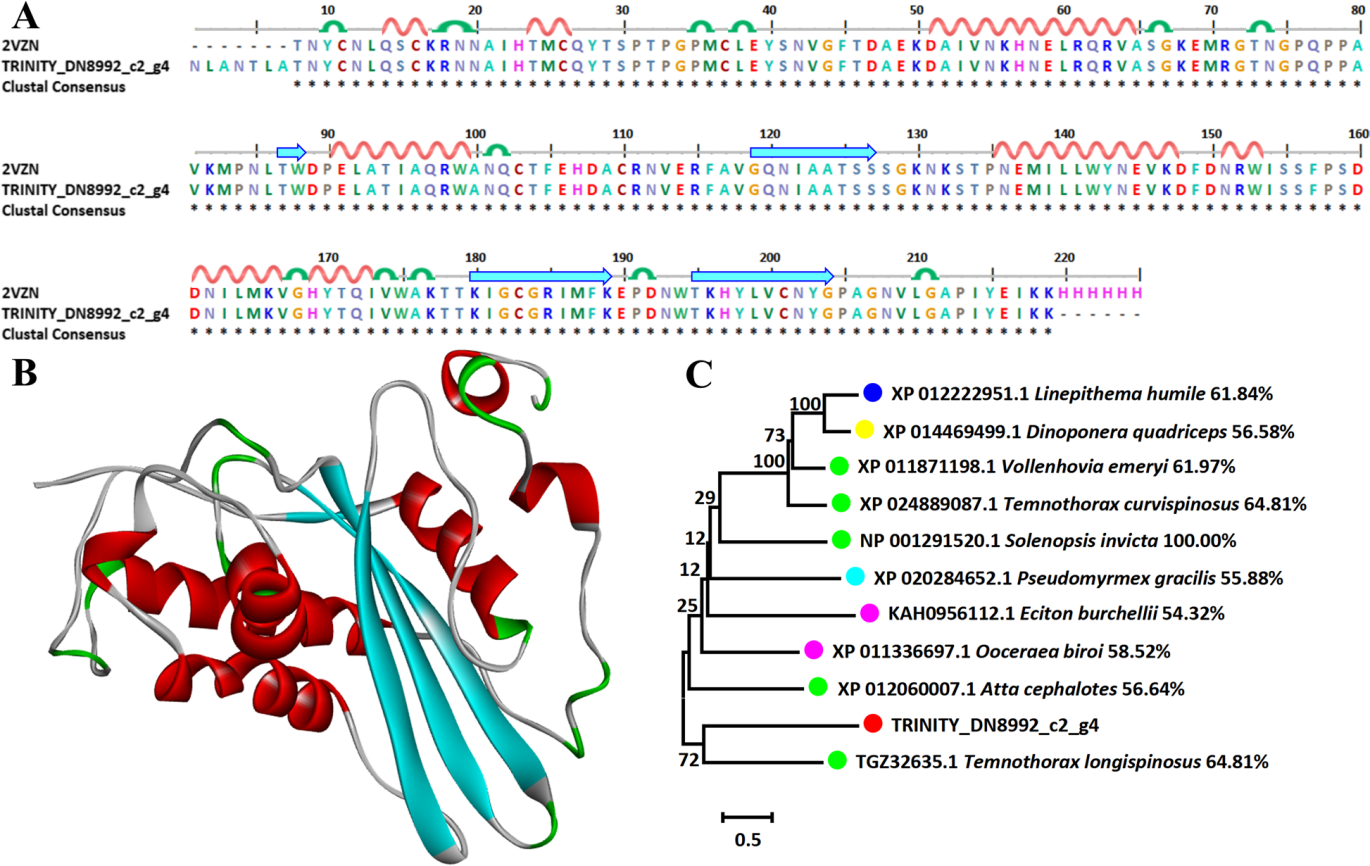
Sequence alignment, 3D modeling and phylogenetic analysis of Venom allergen 3 toxin. **A** Putative sequence TRINITY_DN8992_c2_g4 was aligned with the template (PDB ID: 2VZN). At the bottom of columns, asterisks (*) showed conserved positions, colons (:) showed conserved substitutions and points (.) showed non-conserved substitutions. Grey line, green bend, blue banded arrowhead, and red solenoid represent coil, turn, sheet, and helix, respectively. Different fragments were marked by red framework. **B** 3D modeling was modeled by SWISS-MODEL and viewed by Discovery Studio 4.5. The color grey, green, blue, and red represents coil, turn, sheet, and helix, respectively. The differences of TRINITY_DN8992_c2_g4 compared with the template (PDB ID: 2VZN) were marked with black font. **C** The phylogenetic tree including sequence TRINITY_DN8992_c2_g4 and other ten sequences from different species was constructed by using MEGA 7 with the Neighbor-Joining method. Putative sequence, Myrmicinae, Ponerinae, Dolichoderinae, and Dorylinae were marked as red, green, yellow, dark blue, and pink circles, respectively

The sequence alignment and 3D modeling of dominate toxic component venom allergen 3 were naturally performed with a template (PDB ID: 2VZN). Because the template of TRINITY_DN8992_c2_g4 with 2VZN showed a high similarity of 100.00%, there was no difference in 3D structure (Fig. 5A and B). The construction of phylogenetic tree intuitively showed that the ten species with the highest similarity all belonged to formicidae and may further shed insights on the molecular evolution of venom (Fig. 5C).

### Venom prothrombin activator hopsarin-D

**Fig. 6:**
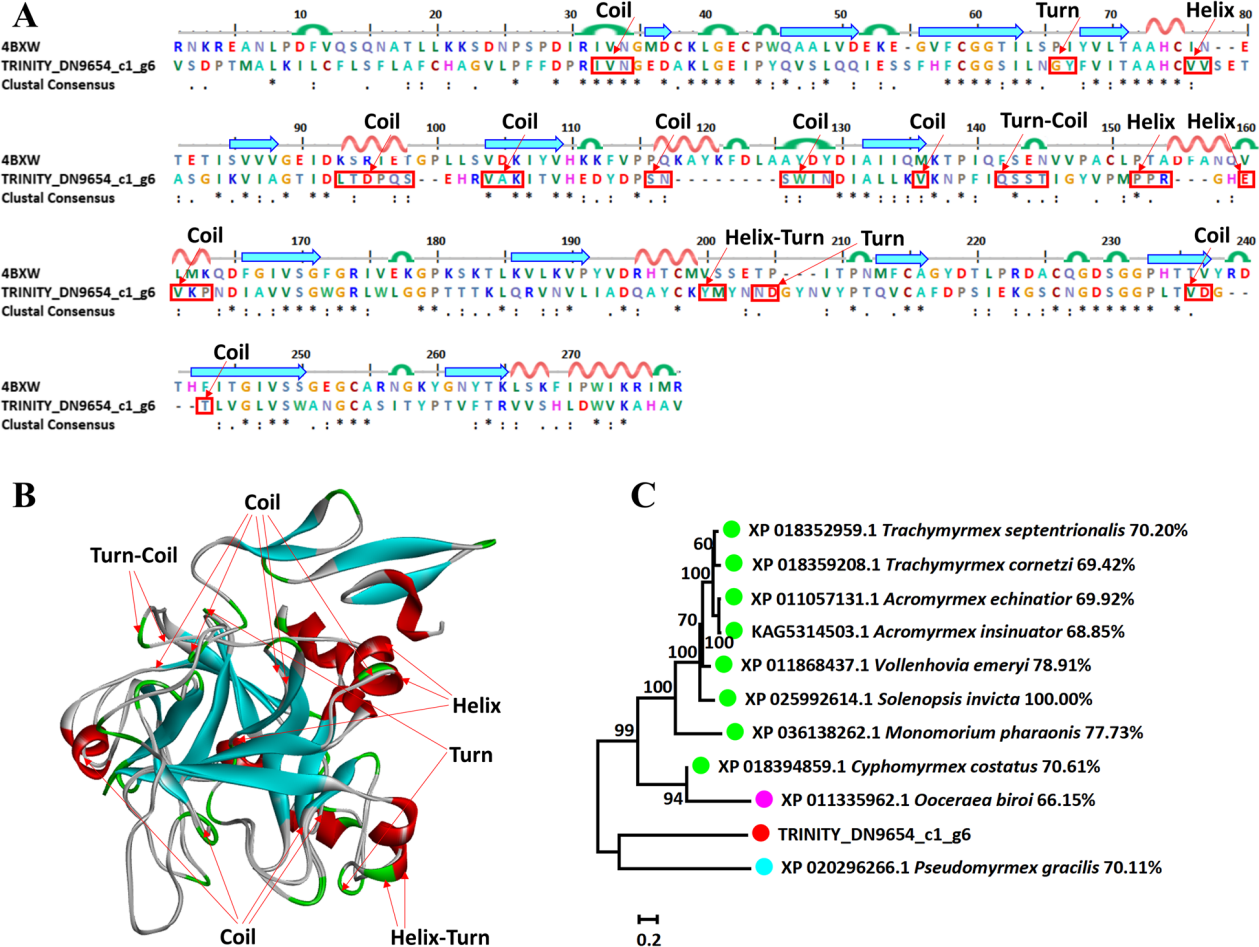
Sequence alignment, 3D modeling and phylogenetic analysis of Venom prothrombin activator hopsarin-D. **A** Putative sequence TRINITY_DN9654_c1_g6 was aligned with the template (PDB ID: 4BXW). At the bottom of columns, asterisks (*) showed conserved positions, colons (:) showed conserved substitutions and points (.) showed non-conserved substitutions. Grey line, green bend, blue banded arrowhead, and red solenoid represent coil, turn, sheet, and helix, respectively. Different fragments were marked by red framework. **B** 3D modeling was modeled by SWISS-MODEL and viewed by Discovery Studio 4.5. The color grey, green, blue, and red represents coil, turn, sheet, and helix, respectively. The differences of TRINITY_DN9654_c1_g6 compared with the template (PDB ID: 4BXW) were marked with black font. **C** The phylogenetic tree including sequence TRINITY_DN9654_c1_g6 and ten other sequences from different species was constructed by using MEGA 7 with the Neighbor-Joining method. Putative sequence, Myrmicinae, Dorylinae, and Pseudomyrmecinae were marked as red, green, pink, and light blue circles, respectively

The sequence alignment and 3D modeling of dominate toxic component venom prothrombin activator hopsarin-D was naturally showed with a template (PDB ID: 4BXW). The template is crystal structure of the prothrombinase complex from the venom of *Pseudonaja Textilis*. TRINITY_DN9654_c1_g6 with 4BXW showed a similarity of 36.28%. The TRINITY_DN9654_c1_g6 had a total of 18 differences with the template, including 10 coils, 4 turns, 4 helixes (Fig. 6A and B). A phylogenetic tree of *S*. *invicta* venom (venom prothrombin activator hopsarin-D) was constructed, and showed different distances between sequence (TRINITY_DN9654_c1_g6) and other species (Fig. 6C).

## Discussion

*Solenopsis invicta* is an invasive species in China. We collected samples from an airport in Guangdong province of China and studied *S*. *invicta* through the transcriptome and proteome. In previous studies on ant toxins, transcriptome had revealed that true venom proteins made up a small fraction of the transcripts being expressed in venom gland tissues (< 1% ~ 5%) [28]. However, a total of 33,231 unigenes and 721 proteins were identified at the transcriptome and proteome levels in this study. After being aligned with the uniprot toxin database, a total of 316 unigenes and 47 proteins were successfully screened. True venom transcripts accounted for 0.95% of the transcripts being expressed in *S*. *invicta* while the toxins accounted for 6.52% of all proteins. Additionally, there were much differences in the number annotation of the overall level and classification function at the transcriptome and proteome levels for the GO and KEGG functional annotation. The reason for these may be associated with the De novo assembly issues or even limitations with the used software to translate the genes into proteins.

*Solenopsis invicta* can sensitize individuals by chelating, touching, and inhaling. Allergens in the *S*. *invicta* venom are the main cause of serious allergic reactions and even death of the individuals attacked [33, 34]. In previous studies [12, 34–40], the most well-known proteins in *S. invicta* venom were potent allergens, including Sol i 1 (venom allergen 1), a phospholipase A/B similar to those reported in wasp venoms; Sol i 2 (venom allergen 2), which apparently seems to be a pheromone binding protein; Sol i 3 (venom allergen 3), a member of the antigen 5/ pathogenesis-related protein; and Sol i 4 (venom allergen 4), a member of a unique protein family of unknown function. Venom allergens 2 ~ 5 were screened in the transcriptome and only venom allergen 3 was found in the proteome, which may be owing to the modification of post-transcription or the limitations of experimental manipulation and sequencing techniques. Dos Santos Pinto et al*.* [41] used the method that combines gel-based and gel-free proteomic strategies to assign the proteomic profile of the venom from *S*. *invicta*, which permitted the identification of 46 proteins. However, our new method effectively screened a total of 316 unigenes and 47 proteins and expanded the toxin databases of *S*. *invicta* venom through comparison with the uniprot toxin database, among them, some we discussed were different from dos Santos Pinto et al*.* [41]. They mainly focused on the structure and function of myotoxin, disintegrin, metalloproteinase, and atrial natriuretic peptide (ANP), while we explored the structure of calglandulin (TRINITY_DN9166_c1_g16), venom allergen 3 (TRINITY_DN8992_c2_g4), and venom prothrombin activator hopsarin-D (TRINITY_DN9654_c1_g6). Additionally, studies revealed the existence of the cross-reactivity among many hymenoptera biocapsules. For instance, cross-reactivity can be shown in all their major proteins of cystic fluid [42]. Sol i 1 protein in *S. invicta* capsule fluid has cross-reactivity with yellow jacket phospholipase [22, 43]. Phospholipases were relatively common in Hymenoptera venoms, occurring mainly as A and B types [44]. Similarly, our work found that *S*. *invicta* toxins contained phospholipase A2, one of the most prevalent proteins in bee toxins [45], which may have contributed to the cross-reactivity shown in Sol i 1 protein of *S*. *invicta* and bees.

The presence of calglandulin (TRINITY_DN9166_c1_g16) was firstly found in *S*. *invicta* venom. This protein was associated with the secretion of toxins from the gland into the venom [46]. Therefore, it may be a potential way to reduce the toxin components in *S*. *invicta* venom by preventing the production of calglandulin, providing a vital foundation for further analysis of the toxins in *S*. *invicta* venom. In addition, a total of seven putative sequences (TRINITY_DN8687_c1_g1, TRINITY_DN8971_c0_g10, TRINITY_DN9070_c0_g3, TRINITY_DN9215_c1_g2, TRINITY_DN9220_c0_g12, TRINITY_DN9692_c1_g4, and TRINITY_DN10314_c0_g3) in the transcriptome and three putative sequences (TRINITY_DN9215_c1_g2, TRINITY_DN9070_c0_g3, and TRINITY_DN9692_c1_g4) in the proteome were identified as serine proteinase-like BMK-CBP in *S*. *invicta* venom. This protein was also only reported in Chinese red scorpion (*Buthus martensii* Karsch) venom, and had no significant hydrolytic activity [47]. We attached and structurally investigated the venom prothrombin activator hopsarin-D (TRINITY_DN9654_c1_g6) which acted as the similar function to mammalian coagulation Fxa [48]. Additionally, compared to other Hymenoptera which contain 70% toxins, *S. invicta* contains only 0.01% toxic proteins [18, 49]. The amount of toxins in *S*. *invicta* venom is extremely low, and the molecular expression of toxins which annotated at the transcriptome and proteome levels were 24,504.26 and 235 respectively. Among them, venom allergens were expressed in 7,746.54 (31.61%) and 14 (5.96%). We confirmed that *S*. *invicta* toxin allergens are the most effective known components that cause sensitization and induce allergic reactions, despite the fact that they were rarely expressed and represented in toxin protein [50].

## Conclusions

In summary, we successfully built the transcriptomic and proteomic databases of *S*. *invicta*. Through comparing with the uniprot toxin database, we successfully screened a total of 316 unigenes and 47 proteins, and predicted the structure of calglandulin (TRINITY_DN9166_c1_g16), venom allergen 3 (TRINITY_DN8992_c2_g4), and venom prothrombin activator hopsarin-D (TRINITY_DN9654_c1_g6). We hope our findings will provide deep insights into *S*. *invicta* toxins.

## Supplementary Information

**Additional file 1: Supplementary Figure 1.** Quality Control of unigenes and proteins.

## Data Availability

All data supporting this study were included in article.

## Abbreviations

GO: Gene Ontology
KEGG: Kyoto Encyclopedia of Genes and Genomes
NR: NCBI nonredundant
LC–MS/MS: Liquid chromatography-mass spectrometry/mass spectrometry
BP: Biological Process
CC: Cell Component
MF: Molecular Function
MW: Molecular weight
PSMs: Peptide spectrum matches
PI: Isoelectric point
