# Supplementary material for: A combined protein toxin screening based on the transcriptome and proteome of Solenopsis invicta
Source: Proteome Sci. 2022 Sep 21;20:15. doi: 10.1186/s12953-022-00197-z (PMC9494847; doi:10.1186/s12953-022-00197-z)
Supplement: Supplementary file 1 — Additional file 1: Supplementary Figure 1. Quality Control of unigenes and proteins. [file 12953_2022_197_MOESM1_ESM.docx]

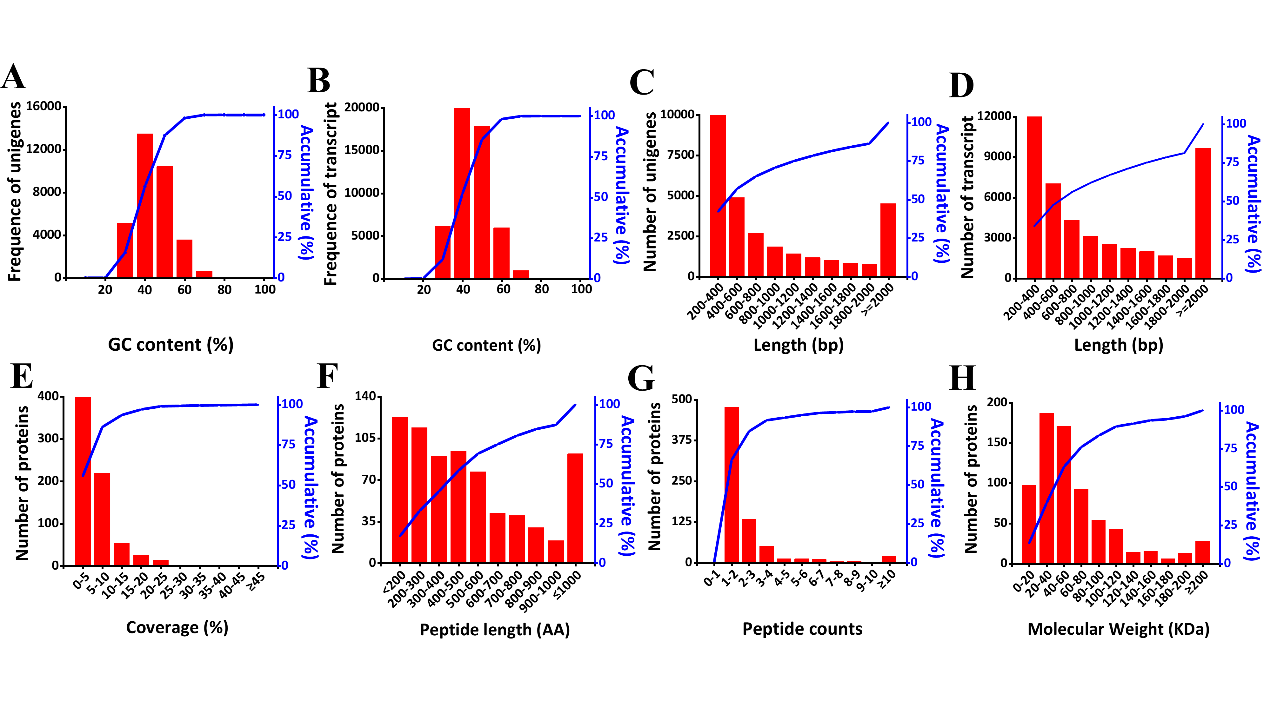


**Supplementary Figure 1. Quality Control of unigenes and proteins. (A)** GC content of unigenes. **(B)** GC content of transcripts. **(C)** The length distribution of unigenes. **(D)** The length distribution of transcripts. **(E)** Protein sequence coverage (%) distribution. **(F)** Peptide length (AA) distribution. **(G)** Peptide counts distribution. **(H)** The relative molecular weight distribution. Y-ordinate represents the cumulative percentage of unigenes and proteins respectively in A ~ H.
